# A case report of a renal anastomosing hemangioma and a literature review: an unusual variant histologically mimicking angiosarcoma

**DOI:** 10.1186/s13000-014-0159-y

**Published:** 2014-08-08

**Authors:** Li-Li Tao, Yi Dai, Weihua Yin, Joan Chen

**Affiliations:** Department of Pathology, Perking University Shenzhen Hospital, Shenzhen, Guangzhou Province PR China; Department of Imaging, Perking University Shenzhen Hospital, Shenzhen, Guangzhou Province PR China; Department of Pathology, Elizabeth hospital, Hongkong, China

**Keywords:** Anasomosing hemangioma, Kidney, Vascular lesions

## Abstract

**Abstract:**

Anastomosing hemangioma, a benign vascular neoplasm histologically simulating angiosarcoma, is newly recognized and has been described primarily in the genitourinary tract. Here, we present a case of renal anastomosing hemangioma originating in the left kidney of a 32-year-old Chinese man with detailed computerized tomography (CT) and enhanced CT image information. The patient had no obvious signs and symptoms. The tumor was incidentally found by color Doppler imaging during a routine heath check-up. Subsequently, a detailed CT and an enhanced CT scan were performed. The tumor was well demarcated, and mahogany brown lesions, which measured 2.6 cm in maximum diameter, were observed. Microscopically, the tumor shows a lobular architecture with alternating cellular areas composed of anastomosing sinusoidal capillary-sized vessels lined by hobnail endothelial cells and edematous, hyaline paucicellular areas. Cytologically, the tumor cells were generally bland and exhibited positivity for CD31 and CD34 immunohistochemically. The patient had good status without evidence of tumor recurrence 21 months after the surgery. We suggest that more attention should be focused on this rare renal hemangioma variant and that it should not be over-diagnosed as a malignance, particularly an angiosarcoma.

**Virtual Slides:**

The virtual slide(s) for this article can be found here: http://www.diagnosticpathology.diagnomx.eu/vs/13000_2014_159

## Background

Primary benign vascular lesions of the kidney are uncommonly encountered in routine surgical pathology practice. A concern is that these lesions can mimic malignancy. Anastomosing hemangioma is a newly recognized variant of capillary hemangioma. Montgomery and Epstein recently described 6 cases of new variants of benign vascular tumors involving the kidneys, perinephric adipose tissue, and testes, which the authors designated as anastomosing hemangiomas [1]. Subsequently, more cases of this novel vascular tumor have been reported. Previously, this tumor seemed to be unique in the genitourinary system [1–3], with a particular proclivity for the kidney. However, more reports showed that, in addition to the genitourinary system, the adrenal gland [4], liver and gastrointestinal tract were potential sites [5]. Microscopically, this tumor is composed of anastomosing sinusoidal capillary-sized vessels and can be over-diagnosed as a malignance, particularly an angiosarcoma [2,3,6]. It is of great clinical importance, for misdiagnosis will results in disastrous consequence to the patient. Anastomosing hemangioma is a benign lesion that can be cured by local excision, while angiosarcoma is a malignant tumor that is capable of metastasis and may not be fully eradicated by localized surgical removal. Until recently, approximately 30 cases of anastomosing hemangiomas had been reported, but rarely with CT image information, and only 1 case was from China. Here, we describe a case of anastomosing hemangioma in a Chinese patient with detailed CT and enhanced CT scan image information. In addition, a brief review of previous cases published in the English-language literature is also discussed.

## Case presentation

### CT scan

**Figure 1:**
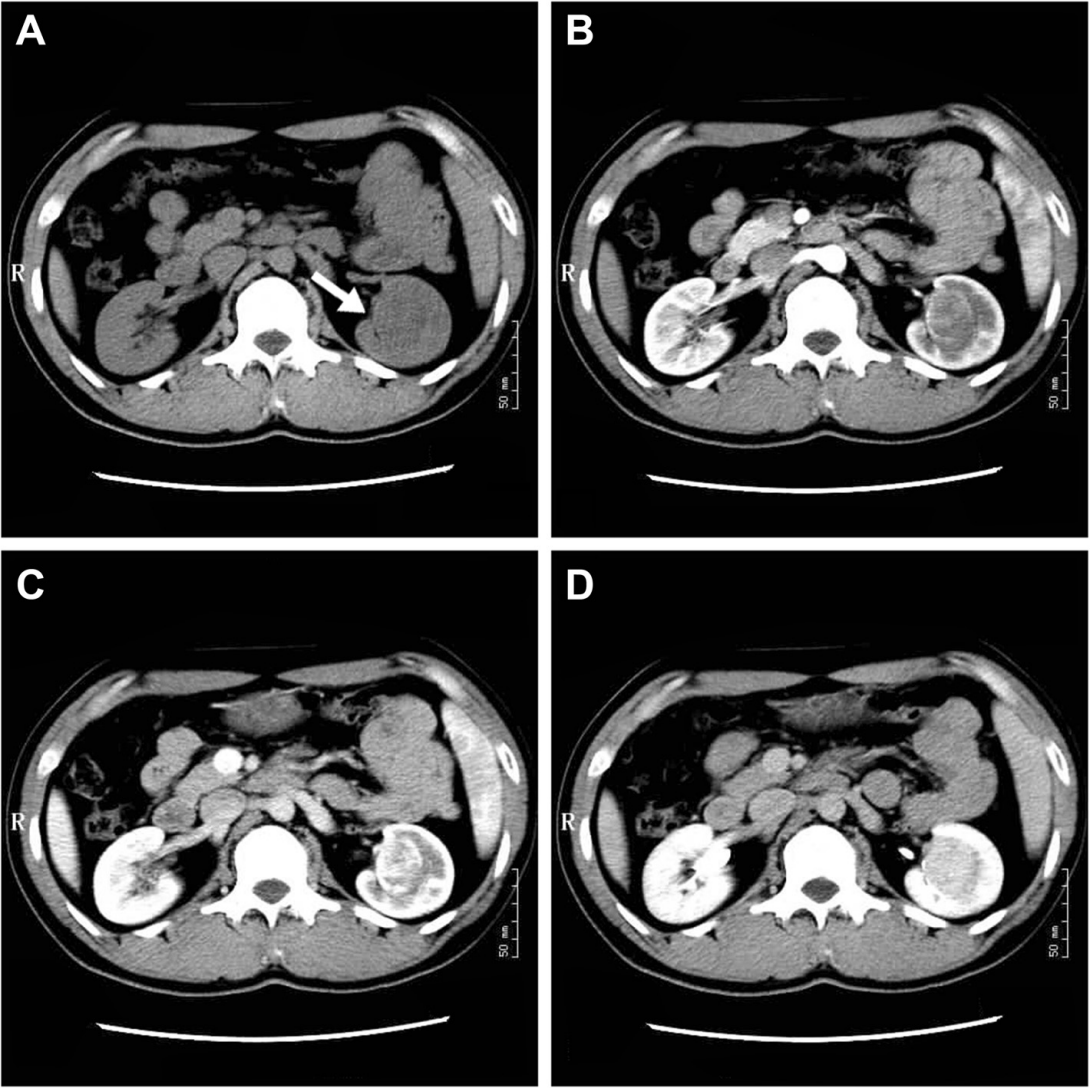
**Unenhanced axial CT and contrast-enhanced CT scans. (A)** Unenhanced axial CT scan. **(B)** Arterial phase of contrast-enhanced CT. **(C)** Venous phase of contrast-enhanced CT. **(D)** Delayed phase of contrast-enhanced CT.

A 32-year-old Chinese male patient with no obviously abnormal medical history was incidentally shown to have a left kidney lesion by color Doppler imaging during a routine heath check-up. The lesion was further detected with an abdominal CT scan. An unenhanced axial CT scan showed a left renal sinus mass with a round, well-circumscribed figure that was approximately 3.4 × 2.7 cm in size (Figure 1A). It appeared to be heterogeneous, and the CT values ranged from 27–35 Hu. The boundary of the lesion showed obviously annular and nodular enhancement with 37–117 Hu in the arterial phase of the contrast-enhanced CT (Figure 1B). In the venous phase, the lesion demonstrated further intense enhancement, which extended to the center with 62–145 Hu CT values (Figure 1C). The lesion showed homogeneously persistent enhancement and a well-circumscribed boundary on delayed images with CT values ranged from 90 to 112 Hu (Figure 1D). The adjacent renal cortex was pressed and thinned, while part of the cortex demonstrated decreasing perfusion.

### HE and immunohistochemical studies

Hematoxylin-eosin (HE) and immunohistochemical staining were performed on formalin-fixed, paraffin-embedded tissues. For immunohistochemical examination, sections were pretreated in a microwave oven and incubated with primary antibodies against CD31 (JC/70A; in use, Maixin, China), CD34 (QBEnd/10; 1:200; Maixin, China), Ki-67 (MIB-2;1:200 Maixin,China), and SMA(1A4, in use; Maixin, China). Immunohistochemistry was performed with an immunohistochemistry autostainer (BenchMArk,XT, ROCHE), with diaminobenzidine as the chromogen and Mayer’s hematoxylin as the counterstain.

## Results

Macroscopic examination demonstrated a round, well-circumscribed mass composed of firm, fleshy, mahogany brown tissue that measured 2.6 cm in maximum diameter, which abutted but did not invade the surrounding renal tissue.

**Figure 2:**
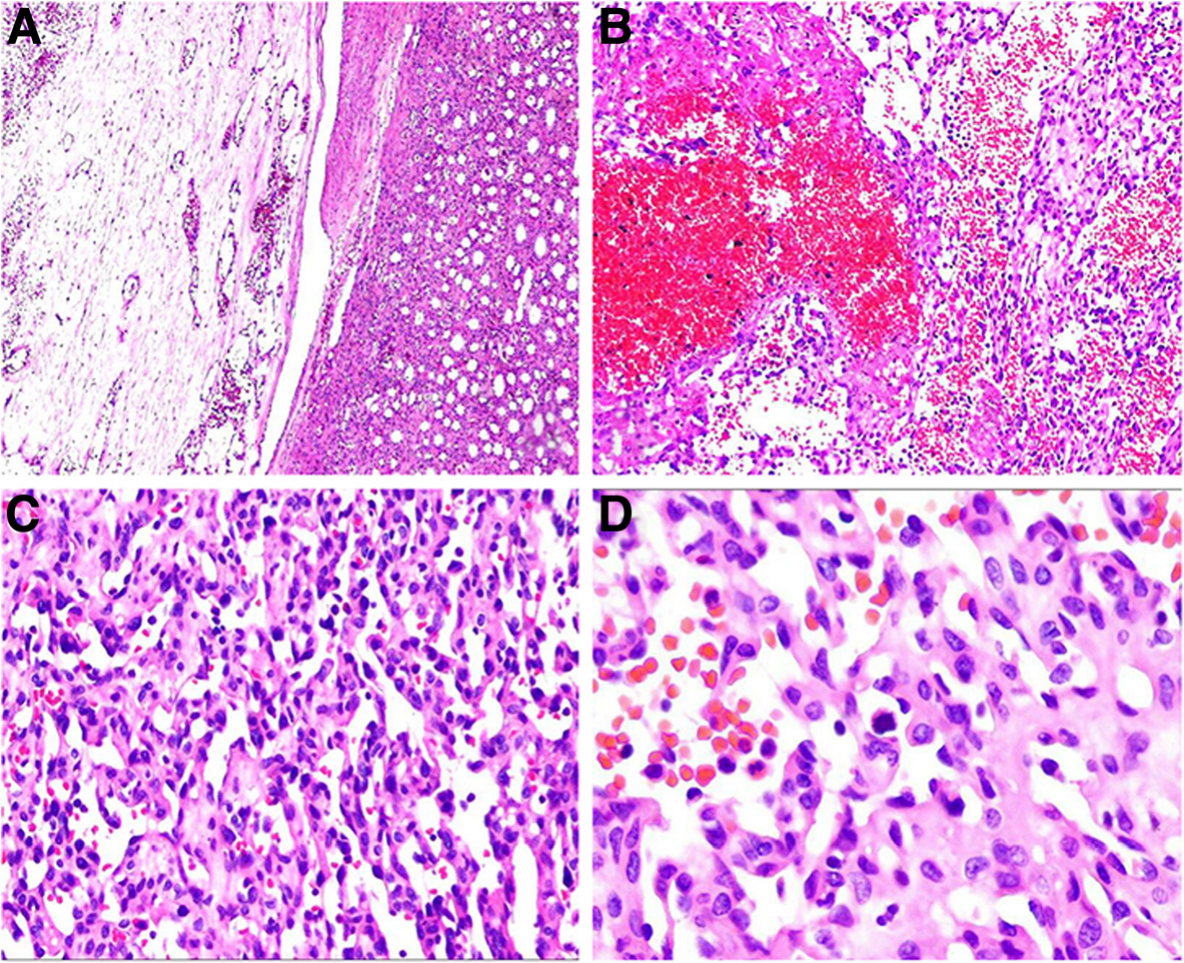
**Histologic features of anastomosing hemangioma. (A)** At low power, the tumor was well demarcated but had no capsula (HE; magnification × 40). **(B)** At higher magnification, the tumor was composed of anastomosing sinusoidal capillary-sized vessels. Scattered intravascular thrombi associated with extravasated, intact RBCs and hemosiderin were seen (HE; magnification × 100). **(C)** At higher power the tumor cells appeared oval to spindle in shape with minimal pleomorphism (HE; magnification × 200). **(D)** Under high magnification, the tumor cells lacked cellular atypia, multilayering of endothelial cells and apoptotic figures or mitotic activity (HE; magnification × 400).

**Figure 3:**
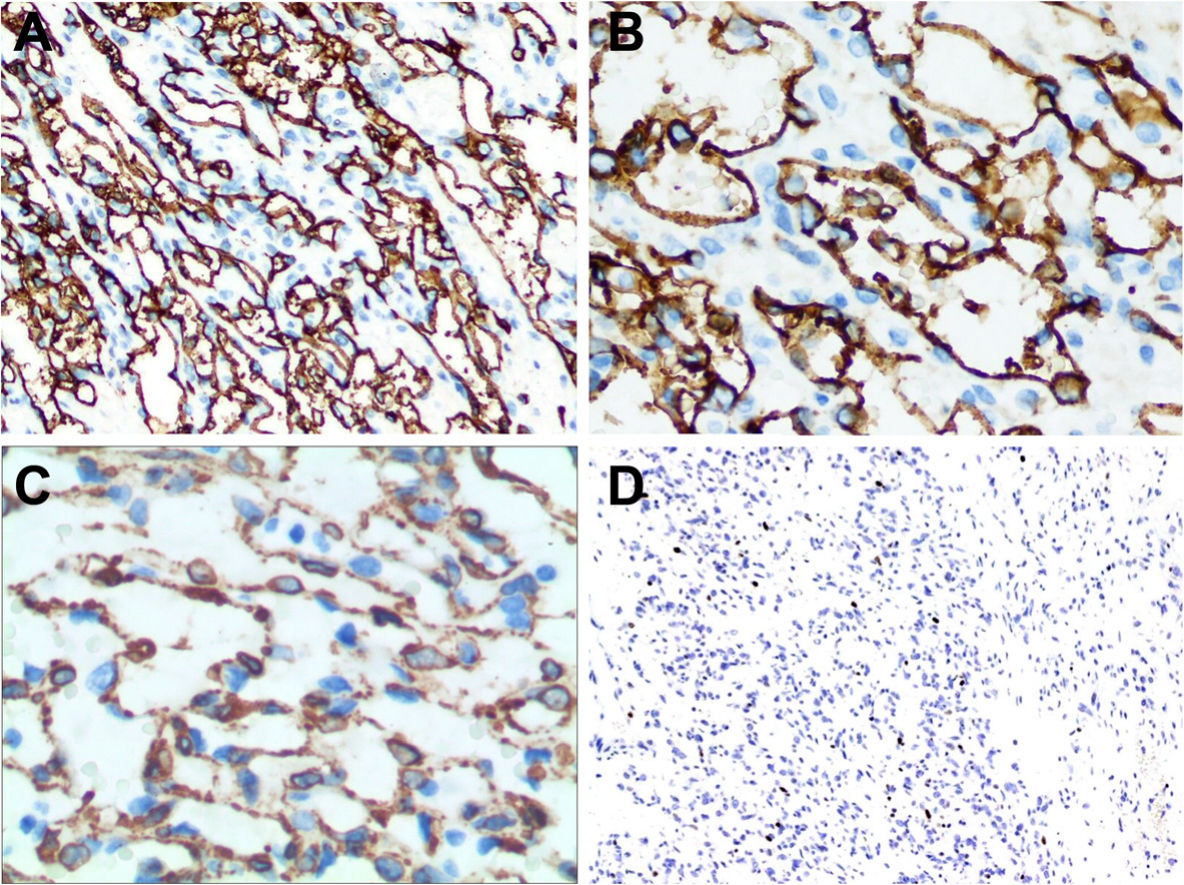
**Immunohistochemical expression of the tumor. (A)** CD 31 (HE; magnification × 100); **(B)** CD34 (HE; magnification × 200); **(C)** SMA (HE; magnification × 400) and **(D)** Ki-67 (HE; magnification × 100).

Microscopically, the tumor was well demarcated but had no capsula. It demonstrated a loosely lobular architecture and showed no evidence of vascular invasion, necrosis or invasion into the perirenal fat or renal tissue. It showed alternating cellular and paucicellular areas at low power. At higher magnification, the tumor was composed of anastomosing sinusoidal capillary-sized vessels. The vessels were lined by hobnail endothelial cells and a flat endothelium focally. Zones of sclerosis and deposition of collagen between the sinusoidal vessels were observed. Cytologically, the tumor cells lacked cellular atypia, multilayering of endothelial cells and apoptotic figures or mitotic activity, although a slight degree of nuclear enlargement was observed. Scant lymphocytes were found, but no plasma cells or acute inflammatory cells were observed (Figure 2A-D). Immunohistochemical studies showed that the tumor cells were diffusely positive for CD31 (Figure 3A) and CD 34 (Figure 3B), and the stroma cells were positive for SMA (Figure 3C). Ki-67 (Figure 3D) expression was approximately 1% positive in tumor cells, which showed a lower proliferation index for the tumor.

The histopathologic appearance and immunophenotypic feature of the tumor were indicative of a vascular tumor; however, as we lacked experience with tumor diagnosis, we could not exclude a high-differentiated angiosarcoma. After consultation with Dr. Joan Chen at the Elizabeth Hospital (Hong Kong, China), the lesion was diagnosed as an anatomosing hemangioma.

## Discussion

Whereas most renal malignancies in adults are epithelial in origin, a small number are mesenchymal. Of these, vascular tumors and tumor-like lesions account for a very small subset [7]. They include a range of benign and malignant lesions, but most of these lesions are benign [3]. Though vascular tumors of the kidney are extremely unusual, hemangiomas of the kidney remain the most common renal vascular tumor [8–10]; in addition, rare cases such as hemangioblastoma was reported in kidney [11].

In 2009, Montgomery and Epstein described 6 cases of new variants of benign vascular tumors involving the kidneys, perinephric adipose tissue, and testes, which were designated as anastomosing hemangiomas [1]. Subsequently, more cases were reported that included the genitourinary system, adrenal gland [4], liver and gastrointestinal tract [5]. Including our case, a total of 31 cases (32 tumors; one patient had two lesions, one in each kidney) presented with a wide age range from 21 years to 83 years (median, 54 years; average, 56.3 years), with most patients within their fifth to sixth decade in age. These tumors showed a slight predilection for females, with a male to female ratio of approximately 1.38:1 (18:13). However, the tumor showed a slight predilection for males if only one kidney was involved [6]. Involved sites included the kidney (61.3%, 19/31), liver (12.9%, 4/31), ovary (9.6%, 3/31), testis (6.5%, 2/31), colon and small bowel (6.5%, 2/31), and adrenal gland (3.2%, 1/31). The tumor size of the 31 cases ranged from 0.1–5.0 cm (median: 2.0 cm, average: 2.1 cm). It seems that the tumor size in the liver (3.35 cm, 4/31) was larger than in the bowel (2.5 cm, 2/31), the kidney (2.1 cm, 20/31; 19 cases, one patient with two tumors), the adrenal gland (2.0 cm, 1/31), and testis (1.6 cm, 2/31). In the ovary, the tumor was the smallest of all tumors in these cases (0.46 cm). The reported clinical manifestations were variable, and some of them were detected incidentally by radiographic investigations for other unrelated reasons; some of the tumors showed a palpable mass in a superficial organ such as the testis; however, it is worthy to note that, in the kidney, approximately 37% (7/19) of the kidney lesions were associated with end-stage renal disease (ESRD; Table 1) [6].

**Table 1:** **Summary of the reported cases of anatomosing hemangioma**

**No.**	**Ref/Year**	**Age/sex**	**Clinical manifestation**	**Site**	**size (cm)**	**Follow-up**
1	1/2009	74/F	Intermittent hematuria	Kidney	1.5	NED, 36 mo
2		75/F	Intermittent hematuria	Kidney	2.0	Unknown
3		65/F	Vague abodominal pain	Perinephric adipose tissue	2.0	NED, 8 mo
4		49/M	NA	Renal hilum	1.3	NED, 12 mo
5		54/M	A palpable mass	Testis	1.5	NED, 8 mo
6		49/M	A palpable mass	Testis	1.7	NED, 12 mo
7	7/2010	56/M	NA	Right Kidney	1.3	Unknown
8		33/F	NA	Left Kidney	3.2	Unknown
9		22/M	ESRD, post transplant	Right Kidney	2.2	NED, 24 mo
10		44/F	NA	Left Kidney	2.0	NED, 72 mo
11		83/F	NA	Left Kidney	3.5	NED, 24 mo
12	2/2011	70/F	Endometrial carcinoma	Ovary, right, cortex	0.2	NED, 25 mo
13		49/F	Benign bilateral serous cysts	Ovary, right, cortex	0.1	NED, 16 mo
14		77/F	Serous cystadenoma	Ovary, left, cortex and medulla	1.1	NED, 32 mo
15		51/F	ESRD, transplant evaluation	Kidney, right, hilum	1	NED, 7 mo
16		39/M	Chronic polycythemia, incidental	Kidney, right, parenchyma	5	NED, 122 mo
17		67/F	Pulmonary embolism, previous knee replacement, incidental	Kidney, left, perinephric	1.2	NED, 6 mo
18		54/F	ESRD, transplant evaluation	Kidney, right and left, parenchyma	1.2;0.6	NED, 3 mo
19	8/2012	49/M	ESRD	Kidney	2.0	NED, 3 mo
20		55/M	ESRD, papillary adenomas	Kidney	0.6	NED, 3 mo
21		45/M	ESRD	Kidney	1.9	NED, 12 mo
22	9/2013	74/M	Lower urinary tract symptoms	Right Kidney	5.0	DUD, 1 mo
23	6/2013	48/M	TACE for HCC, incidental	Right Kidney	2.5	NED, 12 mo
24	5/2013	64/F	Choledochal cyst	Liver, left lobe	3	NED, 67 mo
25		62/F	RCC	Liver, left lobe	2.4	NED, 14 mo
26		70/F	Routine screening	Colon	0.2	NA*
27		68/M	Left flank pain	Small bowel	4.8	NED, 8 mo
28		48/M	Melanoma and seminoma	Liver, right lobe	2	NED, 18 mo
29		71/F	Back pain	Liver, left lobe	6	NED, 96 mo
30	4/2012	49/F	ESRD	Adrenal gland, right	2.0	NA
31	Ours	32/F	Routine screening	Kidney	2.6	NED, 21 mo

*The patient had no follow-up after diagnosis of a benign colon polyp.

M, Male; F, Female; DUD, indicates dead of unrelated disease; ESRD, end-stage renal disease; HCC, hepatocellular carcinoma; NED, no evidence of disease; Ref, reference; TACE, transcatheter arterial chemoembolization; NA, not available.

Radiologically, because of their general small size and nonspecific imaging findings, renal hemangiomas were rarely diagnosed preoperatively [12]. Imaging information about anastomosing hemangiomas in the kidney is limited. In our case, fortunately, we had unenhanced and contrast-enhanced CT image information. The unenhanced axial CT scan showed a left renal sinus mass with a round, well-circumscribed figure that appeared to be heterogeneous. The boundary of the lesion showed an obviously annular and nodular enhancement in the arterial phase of the contrast-enhanced CT scan. In the venous phase, the lesion demonstrated further intense enhancement. The lesion showed homogeneously persistent enhancement and a well-circumscribed boundary on delayed images. The adjacent renal cortex was pressed and thinned, while part of the cortex demonstrated decreasing perfusion.

Macroscopically, anastomosing hemangiomas were 0.1–6 cm in diameter and were well demarcated but always with no capsula. They were accompanied by a mahogany brown spongy appearance, and there was no grossly evident necrosis or vascular invasion [1–7,13].

Microscopically, most lesions were well marginated, but some showed a focally infiltrative pattern and even areas of intravascular extension [1,2,6]. At low power, the lesions demonstrated a loosely lobulated architecture, with alternating cellular zones and hypocellular areas. The cellular areas comprised proliferations of capillary sized vessels in an anastomosing pattern, while the paucicelluar areas comprised loose stroma tissue with elastic thin-walled blood vessels [1,3,7,13,14]. At higher magnification, the vessels were lined by hobnail endothelial cells and a flat endothelium focally. Zones of sclerosis and deposition of collagen between the sinusoidal vessels were observed. Scattered intravascular thrombi in small vessels associated with extravasated, intact RBCs and hemosiderin could be seen. Cytologically, a slight degree of nuclear enlargement was found, but cellular atypia, multilayering of endothelial cells and apoptotic figures or mitotic activity were not detected. Scant lymphocytes were observed, but no plasma cells or acute inflammatory cells were observed. Extramedullary hematopoiesis and intracytoplasmic hyaline globules have been reported in some cases, but not in all [2,3]. It is worth mentioning that intracytoplasmic hyaline globules may facilitate but not confirm a diagnosis of anastomosing hemangioma, nor can it be a mark for differentiating benign or malignant tumors, for it can be observed in many other vascular lesions, including benign and malignant tumors such as papillary hemangiomas [15], pyogenic graulomas [16], Kaposi sarcomas [17], angiosarcomsa [18], and others. It has been interpreted as thanatosomes (secondary lysosomes), which show a positive PAS-D stain in all cases [2,17,18]. Immunohistochemical studies showed that the tumor cells were diffusely positive for CD34 and CD 31, the stroma cells were positive for SMA, and Ki-67 showed low proliferation activity of the tumor cells.

A differential diagnosis occurs both with benign and malignant vascular tumors, benign lesions such as intravascular papillary endothelial hyperplasia [19], atypical florid vascular proliferation [20], which are rarely seen in the kidney; malignant lesions such as epithelioid hemangioendothelioma [21] and angiosarcomas et al. Of all, differential diagnosis with angiosarcoma is of great importance. In our case, the possibility of an angiosarcoma was raised. An angiosarcoma can contain hyaline globules, with the former commonly displaying an anastomosing vascular pattern and hobnail endothelial cells, which could be confused with anastomosing hemangiomas. However, all the reported cases of anastomosing hemangiomas revealed almost no endothelial atypia, an absence of multi-layering and papillary endothelial tufting, and virtually an absence of mitotic activity or apoptotic figures, which are all features that would be unusual for angiosarcomas, which frequently exhibit frankly malignant natures both histologically and clinically [13]. Immunohistochemistry for SMA is helpful. It was found that SMA was positive in the myxoid supporting stroma cells of the anastomosing hemangioma, but negative in angiosarcomas.

All of the reported cases were treated with surgery. Of the 31 cases, follow-up information was available for 26 patients [1–7,13]. Except for one patient who died of unrelated disease, no patients experienced tumor recurrences or metastases. However, many of these cases were recently recognized as having somewhat limited follow-up availability. Long-term surveillance of more cases is warranted to arrive at any definitive conclusion on expected biological behavior.

## Conclusion

In conclusion, we presented a case of anastomosing hemangioma, a recently described variant of a capillary hemangioma in the kidney, with detailed CT and enhanced CT scan image information. In addition, we summarized the clinicopathologic features of all 32 such lesions published in the literature. These lesions are characterized by anastomosing vessel proliferation, which could lead to a false diagnosis of angiosarcoma. As pathologists, we need to be aware of this subset of hemangiomas and should not interpret these lesions as malignant.

## Consent

Written informed consent was obtained from the patient for publication of this Case Report and any accompanying images. A copy of the written consent is available for review by the Editor-in-Chief of this journal.
